# Supplementary material for: Content-rich biological network constructed by mining PubMed abstracts
Source: BMC Bioinformatics. 2004 Oct 8;5:147. doi: 10.1186/1471-2105-5-147 (PMC528731; doi:10.1186/1471-2105-5-147)
Supplement: Additional File 2 — The original results of the above study (non-essential files are deleted to keep the file size under the limit set by BMC bioinformatics). [file 1471-2105-5-147-S2.bz2 › chilibotAdditionalFile2/dip40/679ID9514722E11088/html/MAUB_MAUA.html]

 


 **MAUB** and **MAUA** 
  
Found 74 abstracts in PubMed, retrieved 40.  
 

 What does Google say? 
 PDF only 
| .edu only 

---

**Interactive relationship** (e.g. stimulation, inhibition, etc)

**Neutral relationship**- The crystal structure of this protein complex is known, and the importance of certain residues on amicyanin in its interaction with MADH  [ **MAUA** ]   [ **MAUB** ]  has been demonstrated by site directed mutagenesis.  Ref: 10913294 Biochemistry, 2000
- The crystal structure of MADH  [ **MAUA** ]   [ **MAUB** ]  reveals that each of these residues participates in hydrogen bonding interactions with other active site residues, TTQ or water.  Ref: 11707614 Protein Eng, 2001
- Phylogenetic analysis of amine dehydrogenases MADH  [ **MAUA** ]   [ **MAUB** ]  and AADH indicated that AADH and MADH  [ **MAUA** ]   [ **MAUB** ]  evolutionarily diverged before separation of proteobacteria into existing subclasses.  Ref: 11495996 Microbiology, 2001
- Another site directed mutant, alpha F55E MADH  [ **MAUA** ]   [ **MAUB** ] , exhibited cation binding properties that were the same as those of the native enzyme, indicating that interactions with the carboxylate of Glu can effectively replace the cation pi interactions with Phe in stabilizing monovalent cation binding to the distal site.  Ref: 11591147 Biochemistry, 2001
- Positively charged amino acid residues on the surface of amicyanin are known to stabilize complex formation with MADH  [ **MAUA** ]   [ **MAUB** ]  and influence the ionic strength dependence of complex formation via electrostatic interactions.  Ref: 9860825 Biochemistry, 1998
- This alphaF55A MADH  [ **MAUA** ]   [ **MAUB** ]  exhibits a 400 fold lower Km value for histamine than does native MADH  [ **MAUA** ]   [ **MAUB** ]  when assayed in solution.  Ref: 11924976 Anal Chem, 2002
- These results demonstrate that the physiologic interaction between MADH  [ **MAUA** ]   [ **MAUB** ]  and amicyanin is stabilized by a combination of ionic and van der Waals interactions.  Ref: 9335529 Biochemistry, 1997
- For each mutant, the majority of the protein that was isolated was inactive and exhibited weaker subunit subunit interactions than native MADH  [ **MAUA** ]   [ **MAUB** ] .  Ref: 12641453 Biochemistry, 2003
- Very low levels of active MADH  [ **MAUA** ]   [ **MAUB** ]  were produced by cells expressing D32N, Y119F, Y119E and Y119K MADH  [ **MAUA** ]   [ **MAUB** ]  mutants.  Ref: 11707614 Protein Eng, 2001
- This first MADH  [ **MAUA** ]   [ **MAUB** ]  crystal structure at low ionic strength is compared with the high resolution structure of the related MADH  [ **MAUA** ]   [ **MAUB** ]  from Paracoccus denitrificans recently reported.  Ref: 9748238 J Biol Chem, 1998
- Methylamine dehydrogenase MADH  [ **MAUA** ]   [ **MAUB** ]  and amicyanin form a physiologic complex within which electrons are transferred from the tryptophan tryptophylquinone TTQ cofactor of MADH  [ **MAUA** ]   [ **MAUB** ]  to the type 1 copper of amicyanin.  Ref: 9335529 Biochemistry, 1997
- The biosynthesis of methylamine dehydrogenase MADH  [ **MAUA** ]   [ **MAUB** ]  from Paracoccus denitrificans requires four genes in addition to those that encode the two structural protein subunits,  **mauB**  and  **mauA** .  Ref: 10400578 J Bacteriol, 1999
- The biogenesis of the quinone cofactor tryptophan tryptophylquinone TTQ in methylamine dehydrogenase MADH  [ **MAUA** ]   [ **MAUB** ]  involves the post translational modification of two constitutive Trp residues Trp beta 57 and Trp beta 108 in Paracoccus denitrificans MADH  [ **MAUA** ]   [ **MAUB** ] .  Ref: 12641453 Biochemistry, 2003
- This mutation had no effect on the parameters for methylamine oxidation by MADH  [ **MAUA** ]   [ **MAUB** ] , but significantly affected its interaction with amicyanin.  Ref: 10913294 Biochemistry, 2000
- These mutants grown under MADH  [ **MAUA** ]   [ **MAUB** ]  inducing conditions contained normal levels of the natural electron acceptor amicyanin, but undetectable levels of the beta subunit and low levels of the alpha subunit of MADH  [ **MAUA** ]   [ **MAUB** ] .  Ref: 9403107 Antonie Van Leeuwenhoek, 1997
- The alphaF55A MADH  [ **MAUA** ]   [ **MAUB** ]  immobilized on the electrode exhibited Michaelis Menten behavior in response to varied concentrations of histamine with an approximately 3 fold lower Km value than that exhibited by the immobilized native MADH  [ **MAUA** ]   [ **MAUB** ] .  Ref: 11924976 Anal Chem, 2002
- The alpha D180R mutation also eliminated the ionic strength dependence of the reaction of MADH  [ **MAUA** ]   [ **MAUB** ]  with amicyanin that is observed with wild type MADH  [ **MAUA** ]   [ **MAUB** ] .  Ref: 10913294 Biochemistry, 2000
- Monovalent cations are known to affect the spectral properties of MADH  [ **MAUA** ]   [ **MAUB** ]  and to influence the rate of the gated electron transfer ET reaction from substrate reduced MADH  [ **MAUA** ]   [ **MAUB** ]  to amicyanin.  Ref: 11591147 Biochemistry, 2001
- alphaF55A MADH  [ **MAUA** ]   [ **MAUB** ]  exhibits an increased K d for cation dependent spectral changes and a decreased K d for cation dependent stimulation of the rate of gated ET from N quinol MADH  [ **MAUA** ]   [ **MAUB** ]  to amicyanin.  Ref: 12686138 Biochim Biophys Acta, 2003
- The detection limit for the native MADH  [ **MAUA** ]   [ **MAUB** ]  PPy sensor was approximately 20 microM while the alphaF55A MADH  [ **MAUA** ]   [ **MAUB** ]  PPy sensor exhibited a detection limit of approximately 5 microM, a 4 fold increase compared to the native MADH  [ **MAUA** ]   [ **MAUB** ]  PPy sensor.  Ref: 11924976 Anal Chem, 2002

**Non-interactive relationship** (e.g. studied together, co-existance, homology, etc.)

- The half wave potentials E1 2 of the two azurins and the intermolecular electron transfer rate constants kET from MADH  [ **MAUA** ]   [ **MAUB** ]  Mj to each azurin were determined by cyclic voltammetry.  Ref: 10631606 J Biol Inorg Chem, 1999
- Addition of monovalent cations to the one electron reduced semiquinone form MADH  [ **MAUA** ]   [ **MAUB** ]  results in RR spectral shifts for a number of vibrational modes of the cofactor.  Ref: 8664261 Biochemistry, 1996
- To accomplish the heterologous expression of correctly assembled MADH  [ **MAUA** ]   [ **MAUB** ] , eight genes from the methylamine utilization gene cluster of P. denitrificans, mauFBEDACJG, were placed under the regulatory control of the coxII promoter of Rhodobacter sphaeroides and introduced into R. sphaeroides by using a broad host range vector.  Ref: 10400578 J Bacteriol, 1999
- Site directed mutagenesis has been performed to probe the roles of specific amino acid residues of amicyanin in stabilizing the MADH  [ **MAUA** ]   [ **MAUB** ]  amicyanin complex and determining the observed ionic strength dependence of complex formation.  Ref: 9335529 Biochemistry, 1997
- Our observations illustrate the different modes of H transfer in MADH  [ **MAUA** ]   [ **MAUB** ]  and AADH with fast and slow substrates and highlight the importance of barrier shape in determining reaction rate.  Ref: 11087744 J Biol Chem, 2001
- Preliminary results obtained with MADH  [ **MAUA** ]   [ **MAUB** ]  enzyme electrodes demonstrate the potential for quinoprotein based biosensors.  Ref: 10721104 Adv Exp Med Biol, 1999
- Site directed mutagenesis of either alphaPhe 55 or betaIle 107 a residue in the beta subunit which interacts with alphaPhe 55 converts MADH  [ **MAUA** ]   [ **MAUB** ]  into enzymes with specificities for long chain amines, amylamine or propylamine.  Ref: 12686138 Biochim Biophys Acta, 2003
- These properties are similar to those of the ET reaction of quinol MADH  [ **MAUA** ]   [ **MAUB** ]  and different from those of the gated reaction of N quinol MADH  [ **MAUA** ]   [ **MAUB** ] , whose rate varies considerably with pH and concentrations of specific monovalent cations.  Ref: 9692997 Biochemistry, 1998
- Methylamine dehydrogenase MADH  [ **MAUA** ]   [ **MAUB** ]  may be immobilized in a polypyrrole PPy film on an electrode surface and used as an amperometric sensor for the determination of histamine.  Ref: 11924976 Anal Chem, 2002
- The K m for 0 diaminodecane is 11 microM, compared to 1.2 mM for wild type MADH  [ **MAUA** ]   [ **MAUB** ] .  Ref: 10985763 Biochemistry, 2000
- Methylamine dehydrogenase MADH  [ **MAUA** ]   [ **MAUB** ]  is a tryptophan tryptophylquinone TTQ dependent enzyme that catalyzes the oxidative deamination of primary amines.  Ref: 11591147 Biochemistry, 2001
- The pH dependence of the redox potentials for the oxidized reduced couples of methylamine dehydrogenase MADH  [ **MAUA** ]   [ **MAUB** ]  and aromatic amine dehydrogenase AADH were determined.  Ref: 9603931 J Biol Chem, 1998
- The resulting inactivation is accompanied by the formation of a covalent cross link between the alpha and beta subunits of MADH  [ **MAUA** ]   [ **MAUB** ] .  Ref: 12062431 FEBS Lett, 2002
- Examination of the extent to which disproportionation of the MADH  [ **MAUA** ]   [ **MAUB** ]  and AADH semiquinones occurred as a function of pH revealed that the equilibrium concentration of semiquinone increased with pH.  Ref: 9603931 J Biol Chem, 1998
- This paper reports the first site directed mutagenesis of MADH  [ **MAUA** ]   [ **MAUB** ] , as well as the construction, heterologous expression, and characterization of a six His tagged MADH  [ **MAUA** ]   [ **MAUB** ] . alpha Asp180 of MADH  [ **MAUA** ]   [ **MAUB** ]  was converted to arginine to examine the effect on complex formation with native and mutant amicyanins.  Ref: 10913294 Biochemistry, 2000
- The unusual property of TTQ enzymes of stabilizing the anionic form of the reduced quinol is important for the reaction mechanism of MADH  [ **MAUA** ]   [ **MAUB** ]  because it allows stabilization of physiologically important reaction intermediates.  Ref: 9603931 J Biol Chem, 1998
- The mauF,  **mauB** , mauE D,  **mauA** , mauG, mauL and mauM mutants do not grow on methylamine as a source of carbon and lack methylamine dehydrogenase activity, but they synthesize both the large and the small subunit polypeptides albeit at different ratios.  Ref: 9202457 Microbiology, 1997
- It is now shown that the alphaF55A mutation also increases the rate of the true electron transfer ET reaction from O quinol MADH  [ **MAUA** ]   [ **MAUB** ]  to amicyanin.  Ref: 12437349 Biochemistry, 2002
- This showed that in the complex, the redox linked pH dependent rotation of His95 is hindered because it would cause an overlap of van der Waals radii with residues of MADH  [ **MAUA** ]   [ **MAUB** ] .  Ref: 9860825 Biochemistry, 1998
- One of these, mauG, exhibits sequence similarity to diheme cytochrome c peroxidases and is required for the synthesis of the tryptophan tryptophylquinone TTQ prosthetic group of MADH  [ **MAUA** ]   [ **MAUB** ] .  Ref: 12809487 Biochemistry, 2003
- The crystal structure of alphaF55A MADH  [ **MAUA** ]   [ **MAUB** ]  in complex with its electron acceptors, amicyanin and cytochrome c 551i, has been determined.  Ref: 12437349 Biochemistry, 2002
- betaI107V MADH  [ **MAUA** ]   [ **MAUB** ]  exhibits a strong preference for propylamine, and betaI107N MADH  [ **MAUA** ]   [ **MAUB** ]  exhibits a preference for 1 aminopentane.  Ref: 11733518 J Biol Chem, 2002
- Bond cleavage by MADH  [ **MAUA** ]   [ **MAUB** ]  using a slow substrate ethanolamine occurs with an inflated KIE 14.7 0.2 at 25 degrees C.  Ref: 11087744 J Biol Chem, 2001
- Methylamine dehydrogenase MADH  [ **MAUA** ]   [ **MAUB** ]  is produced by a range of gram negative methylotrophic and autotrophic bacteria, and allows the organisms to utilise methylamine as the sole source of carbon.  Ref: 12686162 Biochim Biophys Acta, 2003
- In this study, site directed mutagenesis of MADH  [ **MAUA** ]   [ **MAUB** ] , kinetic data, and thermodynamic analysis are used to probe the molecular basis for stabilization of the protein complex by an interprotein salt bridge between Arg99 of amicyanin and Asp180 of the alpha subunit of MADH  [ **MAUA** ]   [ **MAUB** ] .  Ref: 10913294 Biochemistry, 2000
- Site directed mutagenesis studies have revealed several roles for this residue in catalysis and electron transfer ET by MADH  [ **MAUA** ]   [ **MAUB** ] .  Ref: 12686138 Biochim Biophys Acta, 2003
- Tryptophan tryptophylquinone TTQ, the cofactor of methylamine dehydrogenase MADH  [ **MAUA** ]   [ **MAUB** ] , is formed by covalent cross linking of two tryptophan residues and incorporation of two oxygen atoms into one of the indole rings to form a quinone.  Ref: 10721104 Adv Exp Med Biol, 1999
- C H bond breakage by tryptophan tryptophylquinone TTQ dependent methylamine dehydrogenase MADH  [ **MAUA** ]   [ **MAUB** ]  occurs by vibrationally assisted tunneling Basran, J.  Ref: 11087744 J Biol Chem, 2001
- Comparison of the 15N NMR spectra of the aminoquinol TTQ intermediates of AADH and methylamine dehydrogenase MADH  [ **MAUA** ]   [ **MAUB** ]  revealed that the substrate derived aminoquinol N of AADH and MADH  [ **MAUA** ]   [ **MAUB** ]  exhibited distinct 15N chemical shifts which are separated by approx.  Ref: 9494080 Biochem J, 1998
- With regard to H transfer, studies with MADH  [ **MAUA** ]   [ **MAUB** ]  have provided the first example of ground state tunnelling of hydrogen driven by the natural, thermally activated, low frequency motions of the enzyme molecule.  Ref: 10830100 Biochem Soc Trans, 1999
- Methylamine dehydrogenase MADH  [ **MAUA** ]   [ **MAUB** ]  and amicyanin form a physiologic complex which is required for interprotein electron transfer.  Ref: 10913294 Biochemistry, 2000
- In contrast, no detectable O semiquinone accumulates during the two sequential one electron oxidations of the O quinol form of MADH  [ **MAUA** ]   [ **MAUB** ]  by amicyanin.  Ref: 8688431 Biochemistry, 1996
- An N semiquinone, which retains the substrate derived N, is the intermediate during the two sequential one electron oxidations of N quinol MADH  [ **MAUA** ]   [ **MAUB** ]  by its physiologic electron acceptor, amicyanin.  Ref: 9692997 Biochemistry, 1998
- To complete the catalytic cycle, MADH  [ **MAUA** ]   [ **MAUB** ]  is reoxidised via an electron transfer ET chain.  Ref: 12686162 Biochim Biophys Acta, 2003
- strain J gives rise to two azurins Az isol and Az iso2 with methylamine dehydrogenase MADH  [ **MAUA** ]   [ **MAUB** ]  Mj.  Ref: 10631606 J Biol Inorg Chem, 1999
- Thus, it has been possible to create forms of MADH  [ **MAUA** ]   [ **MAUB** ]  that exhibit a preference for amines with carbon chain lengths of one, three, five, or seven carbons.  Ref: 11733518 J Biol Chem, 2002
- The two electron oxidation of tryptophan tryptophylquinone TTQ in substrate reduced methylamine dehydrogenase MADH  [ **MAUA** ]   [ **MAUB** ]  by amicyanin is known to proceed via an N semiquinone intermediate in which the substrate derived amino group remains covalently attached to TTQ Bishop, G.  Ref: 10200175 Biochemistry, 1999
- These results also provide good evidence in favor of an aminotransferase mechanism, as opposed to an imine elimination mechanism, for the reaction of MADH  [ **MAUA** ]   [ **MAUB** ]  with substrate methylamine.  Ref: 8688431 Biochemistry, 1996
- Electron transfer ET from N quinol MADH  [ **MAUA** ]   [ **MAUB** ]  to amicyanin is gated by the deprotonation of the substrate derived amino group on TTQ in the enzyme active site.  Ref: 9692997 Biochemistry, 1998
- We also describe the first structures at 1.95 to 2.15 A resolution of an MADH  [ **MAUA** ]   [ **MAUB** ]  in the substrate reduced form and in the presence of trimethylamine and of cesium, two competitive inhibitors.  Ref: 9748238 J Biol Chem, 1998
- Methylamine dehydrogenase MADH  [ **MAUA** ]   [ **MAUB** ]  has been immobilized in a polpyrrole PPy film on an electrode surface and used as an amine sensor for the determination of primary amines.  Ref: 10845365 Anal Chem, 2000
- Only the T122S MADH  [ **MAUA** ]   [ **MAUB** ]  mutant was produced at near wild type levels.  Ref: 11707614 Protein Eng, 2001
- Rapid scanning stopped flow spectroscopy and global kinetic analysis are used to demonstrate that N semiquinone is a true physiologic reaction intermediate which accumulates during the two sequential one electron oxidations of N quinol MADH  [ **MAUA** ]   [ **MAUB** ]  by amicyanin.  Ref: 8688431 Biochemistry, 1996
- A model is also proposed to describe the sequence of events that leads to stable complex formation between MADH  [ **MAUA** ]   [ **MAUB** ]  and amicyanin.  Ref: 10913294 Biochemistry, 2000
- Possible roles for these amino acid residues in stabilizing unusual structural features of the MADH  [ **MAUA** ]   [ **MAUB** ]  beta subunit, protein folding and TTQ biosynthesis are discussed.  Ref: 11707614 Protein Eng, 2001
- The quinoprotein methylamine dehydrogenase MADH  [ **MAUA** ]   [ **MAUB** ] , type I copper protein amicyanin, and cytochrome c 551i form a complex within which interprotein electron transfer occurs.  Ref: 9860825 Biochemistry, 1998
- Methylamine dehydrogenase MADH  [ **MAUA** ]   [ **MAUB** ]  possesses an alpha 2 beta 2 structure with each smaller beta subunit possessing a tryptophan tryptophylquinone TTQ prosthetic group.  Ref: 12437349 Biochemistry, 2002
- Native MADH  [ **MAUA** ]   [ **MAUB** ]  exhibits a strong preference for methylamine over longer carbon chain amines.  Ref: 11733518 J Biol Chem, 2002
- The rates of the ET reactions of these three different redox forms of MADH  [ **MAUA** ]   [ **MAUB** ]  exhibit a DeltaG degrees dependence which is predicted by Marcus theory.  Ref: 9692997 Biochemistry, 1998
- The aauB and aauA genes encode the periplasmic aromatic amine dehydrogenase AADH large and small subunit polypeptides, respectively, and were homologous to  **mauB**  and  **mauA** , the genes for the large and small subunits of methylamine dehydrogenase MADH  [ **MAUA** ]   [ **MAUB** ] .  Ref: 11495996 Microbiology, 2001
- ET from N quinol MADH  [ **MAUA** ]   [ **MAUB** ]  to amicyanin is gated by the transfer of a solvent exchangeable proton Bishop, G.  Ref: 9354627 Biochemistry, 1997
- It is possible, in vitro, to generate unmodified O quinol and O semiquinone forms of MADH  [ **MAUA** ]   [ **MAUB** ]  with dithionite, as well as an N semiquinone form which contains a substrate derived nitrogen.  Ref: 8688431 Biochemistry, 1996
- Site directed mutagenesis was used to alter active site residues of methylamine dehydrogenase MADH  [ **MAUA** ]   [ **MAUB** ]  from Paracoccus denitrificans.  Ref: 11707614 Protein Eng, 2001
- These data indicate that the intermediate which accumulates during the complete oxidation of substrate reduced N quinol MADH  [ **MAUA** ]   [ **MAUB** ]  is not the O semiquinone, but the more slowly reacting N semiquinone,.  Ref: 8688431 Biochemistry, 1996
- The reduction of MADH  [ **MAUA** ]   [ **MAUB** ]  levels was determined by assays of activity and Western blots of crude extracts with antisera specific for the MADH  [ **MAUA** ]   [ **MAUB** ]  beta subunit.  Ref: 11707614 Protein Eng, 2001
- Various monovalent cations influence the enzymatic activity and the spectroscopic properties of methylamine dehydrogenase MADH  [ **MAUA** ]   [ **MAUB** ] .  Ref: 9748238 J Biol Chem, 1998
- Mutants in mauF,  **mauB** , mauE D,  **mauA** , mauG, mauL and mauM were identified.  Ref: 9202457 Microbiology, 1997
- The spectral properties of the indole quinones in H2O approach the corresponding values in Thiobacillus versutus MADH  [ **MAUA** ]   [ **MAUB** ]  C==O stretch at 1612 cm 1, lamdamax at 440mm and are indicative of strongly hydrogen bonding of the C==O and NH groups of the cofactor in the native enzyme.  Ref: 8664261 Biochemistry, 1996
- In contrast, with alphaF55E MADH  [ **MAUA** ]   [ **MAUB** ]  cross linking of subunits is observed.  Ref: 12062431 FEBS Lett, 2002
- To further modulate the substrate specificity of MADH  [ **MAUA** ]   [ **MAUB** ] , the side chain of alphaPhe 55 was repositioned by site directed mutagenesis of residue betaIle 107.  Ref: 11733518 J Biol Chem, 2002
- Phe 97 is not a part of the predicted pathway for electron transfer from the tryptophan tryptophylquinone cofactor of MADH  [ **MAUA** ]   [ **MAUB** ]  to the copper of amicyanin.  Ref: 9585551 Biochemistry, 1998
- It was also shown that the oxidation reduction midpoint potential for AADH is 20 mV less positive than that of MADH  [ **MAUA** ]   [ **MAUB** ]  over the range of pH values that was studied.  Ref: 9603931 J Biol Chem, 1998
- The tryptophan tryptophylquinone TTQ cofactor of methylamine dehydrogenase MADH  [ **MAUA** ]   [ **MAUB** ]  is covalently modified by substrate derived nitrogen during its two electron reduction by methylamine to form an aminoquinol N quinol.  Ref: 9692997 Biochemistry, 1998
- The structure of reduced amicyanin at pH 4.4, with His95 protonated and flipped, was modeled into the structure of the complex of oxidized amicyanin with MADH  [ **MAUA** ]   [ **MAUB** ] .  Ref: 9860825 Biochemistry, 1998
- MADH  [ **MAUA** ]   [ **MAUB** ]  converts primary amines to their corresponding aldehydes plus ammonia.  Ref: 10721104 Adv Exp Med Biol, 1999
- W3A1 methylamine dehydrogenase MADH  [ **MAUA** ]   [ **MAUB** ]  has been studied by stopped flow spectroscopy.  Ref: 10074378 Biochemistry, 1999
- These results also indicate that the orientation of MADH  [ **MAUA** ]   [ **MAUB** ]  and amicyanin when they react with each other in solution is the same as the orientation of the proteins which is seen in the structure of the crystallized protein complex.  Ref: 9335529 Biochemistry, 1997
- The tryptophan tryptophylquinone TTQ linked methylamine dehydrogenase MADH  [ **MAUA** ]   [ **MAUB** ]  is relatively inert towards O2 in its reduced form.  Ref: 9630684 Biochim Biophys Acta, 1998
- Interestingly, the inverse mutation pair of alpha D180R MADH  [ **MAUA** ]   [ **MAUB** ]  and R99D amicyanin did not restore the favorable salt bridge, but instead disrupted complex formation much more severely than did either individual mutation.  Ref: 10913294 Biochemistry, 2000
- Marcus analysis of the reaction reveals that the ET reaction from the N semiquinone MADH  [ **MAUA** ]   [ **MAUB** ]  to amicyanin exhibits the same reorganizational energy and electronic coupling as do the ET reactions of the dithionite reduced O quinol and O semiquinone forms.  Ref: 9692997 Biochemistry, 1998
- These results also provide the foundation for future studies on the molecular mechanisms of MADH  [ **MAUA** ]   [ **MAUB** ]  and TTQ biosynthesis, as well as a system for performing site directed mutagenesis of the MADH  [ **MAUA** ]   [ **MAUB** ]  gene and other mau genes.  Ref: 10400578 J Bacteriol, 1999
- These results indicate that mauM and mauN are not required for MADH  [ **MAUA** ]   [ **MAUB** ]  or TTQ biosynthesis.  Ref: 10400578 J Bacteriol, 1999
- These properties are in marked contrast with those of MADH  [ **MAUA** ]   [ **MAUB** ] , which shows high specificity to amicyanin.  Ref: 12686147 Biochim Biophys Acta, 2003
- Conversion of alphaPhe55 to alanine dramatically alters the substrate preference of MADH  [ **MAUA** ]   [ **MAUB** ] .  Ref: 10985763 Biochemistry, 2000
- These findings suggest that Az iso2 is a novel azurin and perhaps functions as an electron acceptor for MADH  [ **MAUA** ]   [ **MAUB** ] .  Ref: 10631606 J Biol Inorg Chem, 1999
- Addition of monovalent cations NH4, Cs, and CH3 3NH to MADH  [ **MAUA** ]   [ **MAUB** ]  causes further increases in the lamdamax and decreases in the frequency of the C==O stretch 1590 cm 1 with CH3 3NH.  Ref: 8664261 Biochemistry, 1996
- The biosynthesis of methylamine dehydrogenase MADH  [ **MAUA** ]   [ **MAUB** ]  from Paracoccus denitrificans requires four genes in addition to those that encode the two structural protein subunits.  Ref: 12809487 Biochemistry, 2003
- 1994 Science 26 6 90 which is required for the physiologic electron transfer from the tryptophan tryptophylquinone cofactor of MADH  [ **MAUA** ]   [ **MAUB** ]  to heme via the copper center of amicyanin.  Ref: 8679563 Biochemistry, 1996
- It is shown that on exposure to long range UV ultaviolet light, MADH  [ **MAUA** ]   [ **MAUB** ]  is oxidized in the presence of O2 and exhibits substrate dependent steady state oxidase activity.  Ref: 9630684 Biochim Biophys Acta, 1998
- This MADH  [ **MAUA** ]   [ **MAUB** ]  PPy sensor exhibited a response time of less than 3 s.  Ref: 10845365 Anal Chem, 2000
- A stable binary MADH  [ **MAUA** ]   [ **MAUB** ]  amicyanin complex can be formed, and its crystal structure has been solved to 2.5 A resolution by Chen et al.  Ref: 12686162 Biochim Biophys Acta, 2003
- Methylamine dehydrogenase MADH  [ **MAUA** ]   [ **MAUB** ]  is a tryptophan tryptophylquinone TTQ dependent enzyme that catalyzes the oxidative deamination of primary amines.  Ref: 10985763 Biochemistry, 2000
- The presence of 200 mM NH4Cl during the reaction of O quinol MADH  [ **MAUA** ]   [ **MAUB** ]  with amicyanin does not cause any detectable accumulation of a semiquinone species.  Ref: 8688431 Biochemistry, 1996
- An alphaF55A MADH  [ **MAUA** ]   [ **MAUB** ]  PPy sensor was constructed, and its properties were compared to that of the native MADH  [ **MAUA** ]   [ **MAUB** ]  PPy sensor.  Ref: 11924976 Anal Chem, 2002
- Cyclopropylamine is a mechanism based inhibitor of the quinoprotein methylamine dehydrogenase MADH  [ **MAUA** ]   [ **MAUB** ]  from Paracoccus denitrificans.  Ref: 12062431 FEBS Lett, 2002
- In light of these results, the usually accepted steady state reaction mechanism of MADH  [ **MAUA** ]   [ **MAUB** ]  is revised and clarified to indicate that reactions of the quinone form of TTQ are side reactions of the normal catalytic pathway.  Ref: 10200175 Biochemistry, 1999
- Sequence comparison further indicates that MadH  [ **MAUA** ]   [ **MAUB** ]  could be involved in the acetylation of the phosphoribosyl dephospho CoA prosthetic group and MadG could be involved in its biosynthesis.  Ref: 9128730 Eur J Biochem, 1997
- The light dependent oxidation of MADH  [ **MAUA** ]   [ **MAUB** ]  proceeds via a semiquinone intermediate which accumulates to near stoichiometric levels.  Ref: 9630684 Biochim Biophys Acta, 1998
- The Y119F and D32N mutants were purified from cell extracts and found to be significantly less stable than wild type MADH  [ **MAUA** ]   [ **MAUB** ] .  Ref: 11707614 Protein Eng, 2001
- Within the methylamine dehydrogenase MADH  [ **MAUA** ]   [ **MAUB** ]  amicyanin protein complex, long range intermolecular electron transfer ET occurs between tryptophan tryptophylquinone TTQ of MADH  [ **MAUA** ]   [ **MAUB** ]  and the type I copper of amicyanin.  Ref: 9354627 Biochemistry, 1997
- Two putative monovalent cation binding sites in MADH  [ **MAUA** ]   [ **MAUB** ]  have been identified by X ray crystallography Labesse, G.  Ref: 11591147 Biochemistry, 2001
- Using site directed mutagenesis, phenylalanine 55 on the alpha subunit of MADH  [ **MAUA** ]   [ **MAUB** ]  was converted to alanine.  Ref: 11924976 Anal Chem, 2002
- Methylamine dehydrogenase MADH  [ **MAUA** ]   [ **MAUB** ]  utilizes its endogenous tryptophan tryptophylquinone TTQ as a cofactor in enzymatic catalysis, with the C6 carbonyl of the quinone implicated as the site of attack by substrates and other nucleophiles.  Ref: 8664261 Biochemistry, 1996
- The PPy film containing MADH  [ **MAUA** ]   [ **MAUB** ]  was formed electrochemically on a gold minielectrode 1 mm diameter in the presence of ferricyanide.  Ref: 10845365 Anal Chem, 2000
- For L85, a hypertropic madH  [ **MAUA** ]   [ **MAUB** ]  mutant, the light growth response action spectrum is very similar to that of WT even though the photogravitropism action spectrum of L85 has been shown previously to be altered in the near UV region.  Ref: 11540649 Planta, 1991
- Relatively conservative mutations which removed the potentially reactive oxygens on the side chains of Thr122, Tyr119, Asp76 and Asp32 each resulted in greatly reduced or undetectable levels of MADH  [ **MAUA** ]   [ **MAUB** ]  production.  Ref: 11707614 Protein Eng, 2001
- Methylamine dehydrogenase MADH  [ **MAUA** ]   [ **MAUB** ] , amicyanin, and cytochrome c 551i are soluble redox proteins that form a complex in solution Chen, L.  Ref: 8679563 Biochemistry, 1996
- A similar construct introduced into Escherichia coli did not produce detectable MADH  [ **MAUA** ]   [ **MAUB** ]  activity or accumulation of the  **mauB**  and  **mauA**  gene products but did lead to synthesizes of amicyanin, the mauC gene product.  Ref: 10400578 J Bacteriol, 1999
- The tryptophan tryptophylquinone TTQ cofactor of methylamine dehydrogenase MADH  [ **MAUA** ]   [ **MAUB** ]  is covalently modified by nitrogen during its two electron reduction by methylamine to form an aminoquinol N quinol.  Ref: 8688431 Biochemistry, 1996
- The heterologous expression of MADH  [ **MAUA** ]   [ **MAUB** ]  was constitutive with respect to carbon source.  Ref: 10400578 J Bacteriol, 1999
- Paracoccus denitrificans produces two primary enzymes for the amine oxidation, tryptophan tryptophylquinone TTQ containing methylamine dehydrogenase MADH  [ **MAUA** ]   [ **MAUB** ]  and quinohemoprotein amine dehydrogenase QH AmDH.  Ref: 12686147 Biochim Biophys Acta, 2003
- The aminoquinol of AADH is less stable against reoxidation than that of MADH  [ **MAUA** ]   [ **MAUB** ] .  Ref: 9494080 Biochem J, 1998
- The recombinant MADH  [ **MAUA** ]   [ **MAUB** ]  was localized exclusively in the periplasm, and its physical, spectroscopic, kinetic and redox properties were indistinguishable from those of the enzyme isolated from P. denitrificans.  Ref: 10400578 J Bacteriol, 1999
- Methylamine dehydrogenase MADH  [ **MAUA** ]   [ **MAUB** ]  is a tryptophan tryptophylquinone dependent enzyme that catalyzes the oxidative deamination of primary amines.  Ref: 11733518 J Biol Chem, 2002
- Synthesis of enzymes involved in methylamine oxidation via methylamine dehydrogenase MADH  [ **MAUA** ]   [ **MAUB** ]  is encoded by genes present in the mau cluster.  Ref: 9403107 Antonie Van Leeuwenhoek, 1997
- This study illustrates the potential utility of R. sphaeroides and the coxII promoter for heterologous expression of complex enzymes such as MADH  [ **MAUA** ]   [ **MAUB** ]  which cannot be expressed in E. coli.  Ref: 10400578 J Bacteriol, 1999
- Potentiometric analysis of amicyanin in complex with MADH  [ **MAUA** ]   [ **MAUB** ]  indicates that its Em value is not pH dependent from pH 6.5 to 8.5, and exhibits an Em value similar to that of free amicyanin at high pH.  Ref: 9860825 Biochemistry, 1998
- The crystal structure of alphaF55A MADH  [ **MAUA** ]   [ **MAUB** ]  has also been determined with phenylhydrazine covalently bound to TTQ in the active site.  Ref: 12437349 Biochemistry, 2002
- Direct dynamics calculations employing hybrid quantum mechanical and molecular mehanical QM MM potentials and molecular dynamics simulation methods have been used to explore the important dynamic role that enzyme structure has on proton transfer in the C H bond breakage of a methylamine substrate by methylamine dehydrogenase MADH  [ **MAUA** ]   [ **MAUB** ] .  Ref: 12555860 Faraday Discuss, 2003
- Enzymatic and electron transfer activities have been studied by polarized absorption spectroscopy in single crystals of both binary and ternary complexes of methylamine dehydrogenase MADH  [ **MAUA** ]   [ **MAUB** ]  with its redox partners.  Ref: 8621571 J Biol Chem, 1996
- Methylamine dehydrogenase MADH  [ **MAUA** ]   [ **MAUB** ]  possesses an alpha 2 beta 2 subunit structure with each smaller beta subunit possessing a tryptophan tryptophylquinone TTQ prosthetic group.  Ref: 12686138 Biochim Biophys Acta, 2003
- Of the native and mutant proteins that were studied, their observed order of affinity for each other was as follows native MADH  [ **MAUA** ]   [ **MAUB** ]  and native amicyanin > native MADH  [ **MAUA** ]   [ **MAUB** ]  and R99D amicyanin > alpha D180R MADH  [ **MAUA** ]   [ **MAUB** ]  and native amicyanin > alpha D180R MADH  [ **MAUA** ]   [ **MAUB** ]  and R99D amicyanin, and alpha D180R MADH  [ **MAUA** ]   [ **MAUB** ]  and R99L amicyanin.  Ref: 10913294 Biochemistry, 2000
- No cross linking is seen with alphaF55A or alphaF55I MADH  [ **MAUA** ]   [ **MAUB** ]  mutants.  Ref: 12062431 FEBS Lett, 2002
- ... ET reactions from dithionite reduced quinol and semiquinone forms of MADH  [ **MAUA** ]   [ **MAUB** ]  are rate limited by the ET event.  Ref: 9692997 Biochemistry, 1998
- This finding suggests that active recombinant MADH  [ **MAUA** ]   [ **MAUB** ]  is not expressed in E. coli because one of the accessory gene products is not functionally expressed.  Ref: 10400578 J Bacteriol, 1999
- No activity or cross reactive protein was detected in extracts of cells expressing D76N, T122A and T122C MADH  [ **MAUA** ]   [ **MAUB** ]  mutants.  Ref: 11707614 Protein Eng, 2001
- Four residues of the beta subunit of MADH  [ **MAUA** ]   [ **MAUB** ]  which are in close proximity to the tryptophan tryptophylquinone TTQ prosthetic group were modified.  Ref: 11707614 Protein Eng, 2001
- Within the crystals, MADH  [ **MAUA** ]   [ **MAUB** ]  oxidizes methylamine, and the electrons are passed from the reduced tryptophan tryptophylquinone TTQ cofactor to the copper of amicyanin and to the heme of cytochrome c551i via amicyanin.  Ref: 8621571 J Biol Chem, 1996
- The immobilized MADH  [ **MAUA** ]   [ **MAUB** ]  on the electrode exhibited Michaelis Menten behavior similar to that of the free enzyme in solution with a Km value of 1.3 mM.  Ref: 10845365 Anal Chem, 2000
- the TTQ semiquinone of AADH was less stable than that of MADH  [ **MAUA** ]   [ **MAUB** ] .  Ref: 9603931 J Biol Chem, 1998
- When alphaPhe 55 is replaced with Ala, this preference is reversed with alphaF55A MADH  [ **MAUA** ]   [ **MAUB** ]  preferring long chain amines with at least seven carbons Zhu, Z.  Ref: 11733518 J Biol Chem, 2002
